# Bedside diagnosis of silent aspiration using mobile dynamic digital radiography: a preliminary study

**DOI:** 10.1007/s00405-024-08785-9

**Published:** 2024-07-08

**Authors:** Yuji Koyama, Yukuo Morohoshi R.A., Tetsuji Ohta SLP, Minoru Toyokura, Katsuhiro Mizuno, Yoshihisa Masakado

**Affiliations:** 1 Department of Rehabilitation Medicine, Tokai University School of Medicine, Isehara, Kanagawa Japan; 2 Department of Radiologic Technology, Tokai University Hospital, Isehara, Kanagawa Japan; 3 Department of Rehabilitation Technology, Tokai University Hachioji Hospital, Hachioji, Tokyo Japan; 4 Department of Rehabilitation Medicine, Tokai University Hachioji Hospital, 1838 Ishikawa-machi, Hachioji, Hachioji, Tokyo 192-0032 Japan

**Keywords:** Mobile dynamic digital radiography, Bedside diagnosis, Silent aspiration, Speech-language pathologists

## Abstract

**Purpose:**

This study aimed to assess reliable options for bedside diagnosis of silent aspiration in the intensive care unit by examining the use of default grayscale images (DGI) obtained using a mobile, general-purpose, radiography system capable of dynamic digital radiography (M-DDR) and inverted grayscale images (IGI) of DGI.

**Methods:**

This cohort study (exploratory and preliminary) involved 18 adult patients (mean age, 89.0 years) for whom a swallowing assessment request was received from their primary physicians. Fifty-six IGI videoclips were evaluated by three specialists using the penetration-aspiration scale (PAS), with the gold standard being the consensus reading of all three specialists. Another three speech-language pathologists (SLPs) assessed 56 DGI and IGI videoclips using the PAS. PAS scores 1 and 2 were classified as normal range, PAS scores 3–5 as pathological laryngeal penetration, and PAS scores 6–8 as aspiration. The correct rates with IGI and DGI were then determined, and the level of agreement of IGI and DGI evaluations was evaluated.

**Results:**

The correct rate of all evaluators was 100% for normal range, 80–100% for pathological laryngeal penetration, and 83–100% for aspiration with IGI and 100% for normal range, 90% for pathological laryngeal penetration, and 83% for aspiration with DGI. The kappa coefficient for IGI and DGI showed almost complete agreement for abnormal conditions.

**Conclusion:**

Dynamic imaging of swallowing 2–5 ml of liquid using M-DDR performed for elderly patients at the bedside showed that aspiration assessments by SLPs obtained from DGI videos immediately after imaging are acceptable.

## Introduction

The mechanisms underlying the occurrence of dysphagia in the intensive care unit (ICU) are not completely understood [1], but factors may include disuse muscle atrophy, sarcopenia, decreased muscle strength associated with malnutrition [2, 3], cognitive disorders that make following instructions difficult [4], gastroesophageal reflux, and lack of breathing and swallowing coordination [5], as well as direct injury from endotracheal and tracheostomy tubes. For endotracheal intubation tubes in particular, changes in sensation from post-extubation laryngeal injury or tubes have been reported [6]. Post-extubation dysphagia (PED) from long-term endotracheal intubation is reported to occur within a wide range, from 3 to 62% of cases [7]. The risk of occurrence increases with increasing duration of intubation [8] and larger diameter of intubation tubes [9, 10]. The rate of occurrence of post-extubation silent aspiration (SA) specifically is reported to be 17–25% [11–13], and the handling of PED may thus be considered important.

The bedside swallowing evaluation (BSE) [14] used by speech-language pathologists (SLPs), post-extubation dysphagia screening (PDS) [15] used by nurses, and the Yale Swallow Protocol (YSP) [16] that can be used by both SLPs and nurses are all well known internationally as non-instrumental assessments that can be performed soon after extubation in the ICU to screen for aspiration risk. These assessments emphasize evaluations using a 3-ounce water swallow test (3 oz. WST) [17], but much remains contentious about aspiration screening with the 3 oz. WST alone [18, 19]. Under- and overestimation of SA is an issue that needs to be addressed, and reliable means of detecting SA at the bedside are needed in the ICU. Instrumental assessments with a videofluoroscopic swallowing study (VFSS) or fiberoptic endoscopic examination of swallowing (FEES) are considered necessary for conclusive proof of SA.

However, performing these diagnostic imaging examinations for all ICU patients is impractical. Issues in the ICU include the burden on the patient of a series of manual procedures using a nasotracheal intubation tube and the risk of patient agitation or bleeding from FEES. With VFSS, multiple personnel including doctors are needed to move the patient to the fluoroscopy room and deal with problems that occur with the connected devices or in the general condition of the patient and other matters. FEES is considered inferior to VFSS for diagnosing SA, but has the advantage of being able to be performed at the bedside in the ICU.

This study therefore focused on elucidating the physiological mechanisms underlying dysphagia and making bedside assessments of aspiration with dynamic X-ray imaging of swallowing rather than VFSS to clarify treatment options based on that mechanism, in an attempt to introduce to the ICU reliable options for SA diagnosis in critically ill patients using dynamic X-ray imaging optimized for this purpose. Although this requires diagnostic imaging by physicians, we felt that image analysis by SLP as a diagnostic aid to the physician would facilitate rapid bedside aspiration assessment.

The purpose of this study was thus to explore the practicality of aspiration assessment from videos obtained at the bedside using a mobile, general-purpose, radiography system capable of dynamic digital radiography and measures to counter any problems that arise.

## Methods

This cohort study (exploratory and preliminary) was approved by the university hospital’s research ethics committee (approval no. 22R-113). Written, informed consent was obtained from all participants prior to enrolment in this study.

The participants were 18 patients (8 men, 10 women) admitted to the hospital between November 1, 2022 and February 28, 2023 who satisfied all the inclusion criteria and none of the exclusion criteria. The inclusion criteria were: (1) age > 21 years; (2) request for swallowing evaluation from the primary care physician; (3) difficulty moving the patient to the fluoroscopy room and/or performing VFSS (e.g., inability to maintain a suitable sitting position in a wheelchair); and (4) stable respiratory status (respiratory rate < 30 breaths/min; saturation of percutaneous oxygen > 90% without supplemental oxygen). The exclusion criteria were: (1) exacerbation of medical condition; (2) severe dysphagia in which swallowing could not be induced with saliva or a small amount of water; (3) inability to maintain attention; or (4) nutritional management with a nasogastric tube or gastrostomy from before hospitalization.

The patients’ mean age was 89.0 (SD 7.3) years for the total cohort, 87.0 (SD 6.9) years for men, and 90.6 (SD 7.4) years for women. The mean body mass index was 20.5 (SD 3.0) kg/m^2^ for the total cohort, 20.5 (SD 2.7) kg/m^2^ for men, and 20.4 (SD 3.4) kg/m^2^ for women. The underlying pathology was pneumonia in 9 patients (aspiration pneumonia, *n* = 6; other, *n* = 3), cerebrovascular disease in 3 patients, renal/urological disease in 3 patients, heart disease in 1 patient, neuromuscular disease in 1 patient, and liver disease in 1 patient.

### Imaging method

Digital dynamic radiography (DDR) was performed using a mobile DDR (M-DDR) system (AeroDR TX m01, Konica Minolta, Tokyo, Japan) and a flat-panel detector (FPD) (AeroDR fine motion, Konica Minolta). M-DDR can capture a maximum of 20 s of dynamic X-ray images at 15 frames per second (fps).

**Fig. 1:**
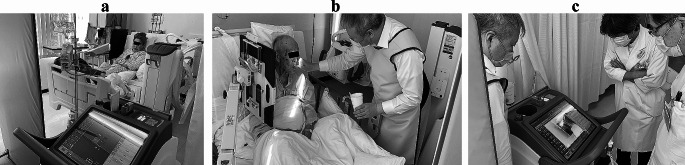
Bedside diagnosis of silent aspiration using mobile dynamic digital radiography. (**a**) The tube is turned toward the window side where no people are present, and the detector is affixed to a holder at the side of the patient. (**b**) Barium swallow is conducted using a 5-ml syringe under lateral imaging. (**c**) Videoclips are reproduced on the M-DDR monitor immediately after imaging to confirm whether aspiration is present

One radiologist, one doctor, and one SLP were involved in the imaging. The participant maintained an upright position in bed with the M-DDR tube facing the window side of the room, where no people were present. A lead partition was placed between the participant and the neighboring patient. An FPD affixed to a mobile detector holder (PLESiO, Obayashi Manufacturing, Saitama, Japan) was brought close to the side of the participant, and the oral cavity, pharynx, larynx, and part of the upper esophagus were included within the imaging range. The distance between the tube and detector was set at 120 cm (Fig. 1a). The initial settings for the imaging system were: 15 fps; tube voltage, 60 kV; tube current, 80 mA; and irradiation time, 5 msec. Tube voltage and tube current conditions were determined by test imaging of the patient. First, lateral imaging was conducted for swallowing 2 ml of liquid (barium sulfate, 38% w/v) using a 5-ml syringe (Fig. 1b). After imaging, videos were immediately reproduced on a 19-inch M-DDR monitor to confirm the presence or absence of aspiration (Fig. 1c). The default grayscale image (DGI) displayed on the M-DDR monitor showed bone and barium fluid as white. If no aspiration was identified, the amount of liquid was increased by 1-ml increments up to a maximum of 5 ml. In other words, data from a maximum of four DGIs were obtained from one patient.

### Evaluation method

Conventional fluoroscopy used in VFSS shows bone and barium fluid as black, whereas DGI, which is standard on the M-DDR monitors, shows bone and barium fluid as white. It was thought that the differences between the two images on the M-DDR monitor should be examined for their potential to make a difference in the determination of pathological laryngeal penetration and aspiration.

**Fig. 2:**
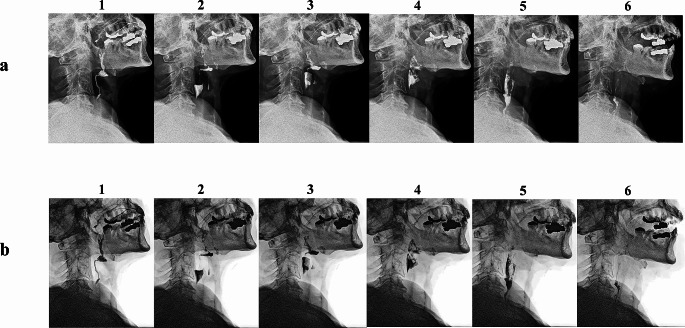
Default grayscale images and inverted grayscale images. From 1 to 6, chronological changes of swallowing. (**a**) Default grayscale images. (**b**) Inverted grayscale images

A total of 59 DGI videos from 18 patients obtained with M-DDR were uploaded to analysis software dedicated to DDR (KINOSIS, Konica Minolta), then black and white were reversed for inverted grayscale image (IGI) videos to create two types of videoclips (Fig. 2). DGI videos that could be examined on the M-DDR monitor showed bone and barium fluid as white, whereas IGI videos showed bone and barium fluid as black, as in conventional X-ray fluoroscopy. All videoclips were saved in MPEG-4 format.

Two physicians and an SLP who specialized in swallowing assessments with more than 30 years of experience evaluated the IGI videoclips using the Penetration-Aspiration Scale (PAS) [20].

1. Material does not enter the airway.
2. Material enters the airway, remains above the vocal folds, and is ejected from the airway.
3. Material enters the airway, remains above the vocal folds, and is not ejected from the airway.
4. Material enters the airway, contacts the vocal folds, and is ejected from the airway.
5. Material enters the airway, contacts the vocal folds, and is not ejected from the airway.
6. Material enters the airway, passes below the vocal folds, and is ejected into the larynx or out of the airway.
7. Material enters the airway, passes below the vocal folds, and is not ejected from the trachea despite effort.
8. Material enters the airway, passes below the vocal folds, and no effort is made to eject.

The three assembled raters reviewed each videoclip frame by frame and excluded three videoclips in which laryngeal penetration and aspiration could not be determined from the images due to video problems. All 56 remaining videoclips from 18 patients were agreed upon by the two physicians and one SLP and were adopted as the gold standard. The 56 videoclips were classified as PAS score 1–8 (1, *n* = 31; 2, *n* = 9; 3, *n* = 4; 4, *n* = 1; 5, *n* = 5; 6, *n* = 2; 7, *n* = 1; and 8, *n* = 3). SA was considered to correspond to PAS score 8. PAS scores of 1 or 2 were classified as within the normal range (*n* = 40), PAS scores of 3–5 were classified as showing pathological laryngeal penetration (*n* = 10), and PAS scores of 6–8 were classified as showing aspiration (*n* = 6).

Fifty-six DGI videoclips were evaluated by three SLPs with at least five years of experience in PAS evaluation who were not involved in the gold standard evaluation. Each evaluator assessed the videoclips as PAS scores 1–8, including slow motion and stop motion with no time limit set for the assessment, and entered and submitted a judgment on a record sheet. At a later date, without revealing that the 56 DGI videoclips had been converted to IGI videoclips, the same SLPs evaluated the 56 randomly reordered IGI videoclips in the same manner, and their judgements were recorded on a recording sheet. Based on the PAS scores of each evaluator, videoclips were classified into three categories: normal range, pathological laryngeal penetration, and aspiration. Images were viewed on a 21.5-inch monitor with a resolution of 4096 × 2304.

1. Correct rate with IGI and DGI

From the IGI and DGI assessments of each evaluator, correct rates for normal range, pathological laryngeal penetration, and aspiration were calculated.

2) Level of agreement of IGI and DGI assessments

The kappa coefficient was used for the level of agreement of IGI and DGI in the evaluations from each of the evaluators.

Statistical analyses were performed using SPSS version 28 software, with kappa coefficients interpreted as follows: 0.21–0.40, fair; 0.41–0.60, moderate; 0.61–0.80, substantial; 0.81–1, almost perfect [21].

## Results

### Correct rates with IGI and DGI

The correct rate for the normal range was 100% (40/40) for all three evaluators with both IGI and DGI. The correct rate for pathological laryngeal penetration was 80% (8/10) for Evaluator A, 100% (10/10) for Evaluator B, and 90% (9/10) for Evaluator C with IGI and 90% (9/10) for Evaluator A, 90% (9/10) for Evaluator B, and 90% (9/10) for Evaluator C with DGI. The correct rate for aspiration was 83% (5/6) for Evaluator A, 83% (5/6) for Evaluator B, and 100% (6/6) for Evaluator C with IGI, and 83% (5/6) for Evaluator A, 83% (5/6) for Evaluator B, and 83% (5/6) for Evaluator C with DGI.

### Level of agreement of IGI and DGI assessments

The level of agreement with IGI and DGI assessments for the normal range was complete agreement for all three evaluators. The kappa coefficients with IGI and DGI for abnormal conditions (pathological laryngeal penetration and aspiration) were 0.885 for Evaluator A, 0.867 for Evaluator B, and 0.881 for Evaluator C, showing almost perfect agreement.

## Discussion

In elucidating the physiological mechanisms underlying dysphagia and clarifying treatment options as a result, VFSS at ≥30 fps is recommended. The use of videos obtained with M-DDR at 15 fps for that same purpose is inappropriate [22].

In this study, the focus was narrowed to aspiration assessments at the bedside, and a preliminary experiment was performed with the idea of introducing to the ICU reliable options for SA diagnosis with dynamic X-ray imaging of swallowing optimized for that purpose.

In cases of laryngeal penetration, when the VFSS of adults is decreased from 30 fps to 15 fps, PAS score 2 may be misjudged as PAS score 1 [23]. In addition, children carry a risk of PAS score 4 being misjudged with the same reduction in frame rate [24]. However, in cases of aspiration, no reports have indicated the possibility that the information necessary for PAS scoring might be completely lost with the same reduction in frame rate, regardless of age. However, little is currently known about the effects of a decreased frame rate on PAS scoring. In the present study, PAS scores were classified into the three categories of “normal range”, “pathological laryngeal penetration”, and “aspiration” in adult patients, and analyses were limited to these categories.

Physicians and SLPs accustomed to VFSS image analysis may prefer IGI in black bone mode to the unfamiliar DGI in white bone mode, which is standard on the M-DDR monitors. Although there are a fair number of reports of the effect of inverting the black and white contrast on evaluations of still images, to the best of our knowledge, there are no reports of the diagnosis of aspiration with videos at 15 fps. Studies have shown that dark characters on a light background (positive polarity) are more legible than light characters on a dark background (negative polarity) [25–27]. In fact, better legibility with decreasing character size is achieved with positive polarity compared to negative polarity [28]. In this case, DGI corresponds to negative polarity and IGI to positive polarity.

The 18 patients for whom bedside evaluation was indicated had a mean age of 89 years, and evaluations by the three SLPs yielded a correct rate of ≥80% for all pathological laryngeal penetration and aspiration with both DGI and IGI. In all evaluated cases of pathological laryngeal penetration and aspiration, almost perfect agreement was obtained between DGI and IGI. A similarly high correct rate and agreement between DGI and IGI were also obtained for patients within the normal range. Thus, inverse processing from DGI to IGI does not seem to be essential when evaluating pathological laryngeal penetration. Furthermore, the SLPs’ determinations of “pathological laryngeal penetration” and “aspiration” appeared to be reliable.

One possible reason for the lack of difference in the diagnosis of aspiration with DGI and IGI is the difference in the black and white contrast for tissue including the larynx and liquid contrast agents, because clearly detecting both is essential for diagnosing aspiration. Though more detailed diagnosis of aspiration remains an issue, the possibility that diagnostic accuracy is heightened by making the diagnosis with a combination of DGI and IGI cannot be ruled out.

Given these findings, assessments by SLPs of the presence or absence of aspiration using DGI videos that can be reproduced immediately after M-DDR are acceptable. This finding suggests that SLPs’ assessments could serve as a diagnostic aid for physicians, and rapid bedside evaluation at the bedside may be feasible.

In our view, the option of this new instrumental swallowing assessment may be beneficial in standardizing transdisciplinary swallowing treatment protocols for critically ill patients.

### Study limitations

Videos that can be checked on an M-DDR monitor can only be checked from recordings, not in real time. With respect to this, live-view images corresponding to 2 fps displayed on an M-DDR monitor after a delay of several seconds were used during imaging. When a judgment could not be made during imaging, 20 s was taken as the time limit for imaging. As a result, all elderly subjects were able to withstand the swallowing assessment with 2–5 ml of liquid.

When performing lateral dynamic X-ray imaging of swallowing in this study, the M-DDR tube was always directed toward the window side where no people were present, and a lead partition was always placed between the participant and the neighboring patient.

Naturally, the addition of an assessment of continuous swallowing of liquid from a cup necessitates an extension of maximum imaging time. Optimizing the imaging conditions for aspiration assessments at the bedside represents a key issue for the future.

## Conclusions

Bedside imaging of elderly patients swallowing 2–5 ml of liquid using M-DDR appears feasible. A correct rate of ≥80% for assessment by SLPs in all cases of pathological laryngeal penetration and aspiration was obtained with DGI, which can be checked on M-DDR, and inverse-processed IGI. Almost perfect agreement was also obtained for assessments from DGI and IGI. Assessment of aspiration using DGI by SLPs obtained immediately after M-DDR appears acceptable.

## Data Availability

Not applicable.

## Abbreviations

SLP: Speech-language pathologist
ICU: Intensive care unit
DGI: Default grayscale images
IGI: Inverted grayscale images
PAS: Penetration-Aspiration Scale
SA: Silent aspiration
VFSS: Videofluoroscopic swallowing study
FEES: Flexible endoscopic evaluation of swallowing
PED: Post-extubation dysphagia
3-oz WST: 3-ounce Water Swallow Test
M-DDR: Mobile-digital dynamic radiography
FPD: Flat-panel detector
fps: Frames per second
